# Integrating gratitude-based interventions with peer education improves outcomes in school-aged children with asthma: A two-phase clinical study

**DOI:** 10.1097/MD.0000000000045008

**Published:** 2025-10-17

**Authors:** Haixin Chen, Xiuming Wei, Lijin Gao, Lijia Liu, Miaomiao Chen, Zhou Junli, Junping Du, Wen Zhang

**Affiliations:** a Department of Pediatrics, The 980th Hospital of the People’s Liberation Army Joint Logistics Support Force, Shijiazhuang, Hebei, China.

**Keywords:** asthma, children, family functioning, gratitude-based interventions, non-randomized controlled trial, peer education, self-management

## Abstract

Childhood asthma remains poorly controlled globally. We aimed to identify social, behavioral, and comorbid factors affecting asthma control and evaluate a novel management strategy combining gratitude-based positive psychology interventions with peer interaction education for asthma self-management. This study employed a sequential two-phase design. Phase 1 comprised a retrospective analysis of clinical data from 152 children with asthma (aged 6–14 years) treated from June 2022 to June 2023 to identify social, behavioral, and comorbid risk factors for poor control. Phase 2 consisted of a prospective non-randomized controlled trial evaluating 120 children allocated to either a control group (CG, n = 60) or research group (RG, n = 60) from December 2023 to December 2024. Allocation occurred through a time-based enrollment process. The RG received an integrated program combining gratitude-based psychological interventions with structured peer interaction components (facilitated child-only small groups with skills rehearsal and mutual symptom monitoring) targeting asthma self-management in addition to standard care. Outcomes included self-management abilities (primary outcome), asthma control, quality of life, family functioning, caregiver psychological parameters, and clinical outcomes. Multivariable analysis from Phase 1 identified key risk factors for poor asthma control including comorbid allergic rhinitis (odds ratio (OR) = 1.23, 95% confidence interval (CI): 0.98–1.67), guardian smoking (OR = 1.36, 95% CI: 1.02–2.17), medication non-adherence (OR = 1.44, 95% CI: 1.12–2.37), low family functioning (OR = 4.59, 95% CI: 1.12–9.33), and absence of health education (OR = 5.13, 95% CI: 1.54–7.80). In Phase 2, the RG demonstrated significantly higher treatment adherence (90% vs 76.7%, *P* = .033), improved asthma control scores (adjusted mean difference: 2.3 points, 95% CI: 1.1–3.5, *P* = .049), enhanced quality of life (adjusted mean difference: 0.6 points, 95% CI: 0.3–0.9, *P* = .018), and better family functioning (*P* = .004) compared to the CG. The RG experienced fewer clinical events with incidence rate ratio of 0.33 (95% CI: 0.12–0.91, *P* = .032) for relapses over 12 months of follow-up. Integrating gratitude-based interventions with peer education is associated with clinically meaningful improvements in self-management, asthma control, and quality of life in school-aged children with asthma.

## 1. Introduction

Bronchial asthma is a common chronic inflammatory disease of the airways, and epidemiological studies have shown that school-age children are at high risk for bronchial asthma, with prevalence rates ranging from 0.12 to 3.41%, with an increasing trend.^[1,2]^ Currently, asthma can be controlled through guideline-based pharmacotherapy plus behavioral supports, though the overall situation of asthma control remains suboptimal. According to surveys, 56.5% of children with asthma in Europe are poorly controlled, while about 20% of children with asthma in China are uncontrolled.^[3–5]^ Moreover, asthmatic children generally tend to present with more than one asthma attack, and the proportion of acute exacerbations in asthmatic children in China is 15.5% per year.^[6]^ Childhood asthma remains a significant global public health challenge.

Several studies have shown that child self-management is an important factor in asthma control, and parents play a key role in child self-management.^[7]^ Additionally, the child’s own factors and environmental factors are also important triggers of asthma.^[8]^ Studies have shown that focusing on the risk factors of asthma in children, health education for asthmatic children and their parents can effectively improve children’s self-management and thus enhance asthma control.^[9]^ Patient education has always been the focus of standardized asthma management. Enhancing patients’ and families’ understanding of asthma disease knowledge, making them proficient in inhalation therapy techniques, and further improving their own awareness and ability to monitor and treat the disease are effective ways to enhance patient and family compliance and improve asthma control.^[10]^ Therefore, effective health education is an important strategy to improve asthma control.

Currently, asthma disease education mainly relies on in-office communication, online multimedia knowledge dissemination, classroom lectures, and a small number of asthma patients’ group activities, which are primarily delivered as “unilateral knowledge instillation.” This approach often reduces the subjective initiative of children and caregivers. Peer interaction education, which involves communication between peer groups, may better mobilize children’s subjective desire to participate in learning and practice. Peer relationships are simple and healthy, and in the context of solidarity and mutual assistance, this educational approach may be more effective in promoting self-management abilities.^[11]^

In addition, asthma is often accompanied by various negative emotions, which can aggravate the condition and reduce quality of life. Therefore, effective psychological intervention is an important strategy to control asthma attacks.^[12]^ Research has revealed that individuals with high levels of gratitude are more likely to focus on positive stimuli and adopt positive coping styles by amplifying positive factors to continuously enhance individual positive psychological experiences, which in turn improves patients’ negative emotions and enhances their sense of well-being.^[13]^ As a theory of positive psychology, the gratitude-based positive psychology emphasizes that the source of positive emotions is mainly derived from evolution, with obvious purpose and adaptability. This theory has been successfully applied in managing malignant tumors and chronic diseases. Some studies have shown that a nursing program based on the gratitude-based positive psychology can increase the level of gratitude, alleviate negative emotions, and improve the quality of life of patients with chronic conditions.^[14]^

Accordingly, this study employed a two-phase design to address the management of school-aged children with asthma. In the first phase, we retrospectively analyzed clinical data to identify key risk factors associated with poor asthma control. Building on these findings, the second phase prospectively evaluated a novel management strategy that organically combines gratitude-based positive psychology with peer interaction education. We assessed the effect of this integrated approach on children’s therapeutic outcomes, self-care abilities, treatment compliance, hospitalization rates, and family functioning, aiming to provide an evidence-based foundation for comprehensive management of school-aged children with asthma.

## 2. Methods

### 2.1. Study design and setting

This investigation employed a sequential two-phase design conducted at the Department of Pediatrics, No. 980 Hospital of the Joint Logistics Support Force. Phase 1 comprised a retrospective analysis of clinical data from children with bronchial asthma treated between June 2022 and June 2023, with the objective of identifying social, behavioral, and comorbid risk factors associated with poor asthma control. Phase 2 consisted of a prospective non-randomized controlled trial conducted from December 2023 to December 2024, evaluating the effectiveness of a novel disease management strategy that combined gratitude-based positive psychology interventions with peer interaction education.

The study was conducted in accordance with the Declaration of Helsinki and approved by the Ethics Committee of No. 980 Hospital of the Joint Logistics Support Force (Approval No. 2023-087). Written informed consent was obtained from the legal guardians of all participants prior to enrollment.

### 2.2. Participants

#### 2.2.1. Phase 1: retrospective analysis

We analyzed data from 152 children with bronchial asthma treated in our hospital from June 2022 to June 2023. Based on asthma control test (ACT) scores, these children were classified into fully controlled (n = 102) and poorly controlled (n = 50) groups. Comprehensive clinical and demographic data were extracted from electronic medical records.

#### 2.2.2. Phase 2: prospective non-randomized controlled trial

For the intervention study, 120 eligible children with bronchial asthma admitted to our hospital from December 2023 to December 2024 were enrolled. A priori power analysis determined that based on pilot data (standard deviation = 4.2 for ACT scores), a sample size of 60 participants per group would provide 85% power to detect a clinically meaningful difference in ACT scores (≥2 points, the minimum clinically important difference (MCID)) with a significance level of 0.05, accounting for an anticipated 15% attrition rate.

Inclusion criteria were: confirmed diagnosis of childhood bronchial asthma based on: recurrent respiratory symptoms (wheeze, shortness of breath, chest tightness, cough), variable expiratory airflow limitation documented by spirometry (≥12% and 200mL improvement in forced expiratory volume in 1 second post-bronchodilator) or peak flow variability > 13%, and physician adjudication according to global initiative for asthma guidelines^[14]^; age 6 to 14 years; treatment according to the global initiative for asthma recommended protocol^[16]^; normal development, stable condition, and adequate cognitive and communication abilities; and provision of signed informed consent by guardians.

Exclusion criteria included: acute exacerbations at enrollment; concurrent severe infectious or medical diseases; other respiratory diseases that could confound assessment; psychiatric disorders; language deficits that would impair participation; or previous enrollment in related studies.

Children were recruited during scheduled admissions for asthma management optimization or from outpatient clinics. Those experiencing acute exacerbations were enrolled only after stabilization (defined as no requirement for supplemental oxygen or frequent bronchodilators for 48 hours).

### 2.3. Group allocation

Participants in Phase 2 were allocated in a 1:1 ratio to either the research group (RG) or control group (CG) using a sequential enrollment process based on hospital admission dates. Children admitted during weeks 1 and 3 of each month were assigned to the RG, while those admitted during weeks 2 and 4 were assigned to the CG. To minimize contamination, groups were assigned to separate ward areas when hospitalized, and social media groups were created separately for each study arm with instructions not to share intervention materials. Enrollment month was included as a covariate in all adjusted analyses to account for potential seasonality effects. Due to the nature of the intervention, complete blinding of participants and care providers was not feasible; however, outcome assessors were blinded to group allocation through a coding system that masked group identity during evaluation.

### 2.4. Data collection

For Phase 1, after obtaining ethics approval, we collected the following data through the hospital’s electronic case query system:

Patient characteristics included age, gender, disease duration, obesity status (defined as BMI ≥ 95th percentile for age and sex), personal history of allergies, presence of allergic rhinitis, and asthma control measured by the ACT.^[15]^ The ACT contains 5 dimensions, each rated from 1 to 5 points (total score 5–25), with scores of 20 to 25 indicating controlled asthma and ≤19 indicating poor control (further subdivided into uncontrolled^[5–15]^ and partially controlled^[16–19]^).

Caregiving information encompassed primary caregiver identity, adherence to glucocorticoid therapy, guardian smoking status, family functioning, caregiver mood, health education status, physical activity level, and follow-up regularity. Phase 1 analysis did not include established clinical predictors such as blood eosinophils, fractional exhaled nitric oxide, or baseline forced expiratory volume in 1 second due to incomplete data availability in retrospective records.

For Phase 2, the same comprehensive baseline data were collected from all 120 participants prior to group allocation using standardized assessment tools administered by trained research assistants.

### 2.5. Outcome measures and scale definitions

All scales and their scoring were defined as follows:

1. **Self-management ability (primary outcome):** Assessed using a validated 34-item scale for children aged 7 to 17 years measuring daily life management, medical disease management, and psychosocial management. Scores range from 34 to 170 (higher scores = better self-management). Categories: high level ≥ 66% of maximum score; intermediate 33 to 66%; low ≤ 33%. Cronbach alpha = 0.866.
2. **Asthma control:** ACT scores (5–25 points; higher = better control; ≥20 = controlled asthma).
3. **Quality of life:** Pediatric asthma quality of life questionnaire (PAQLQ) with 23 items across activities (5), symptoms (10), and emotional function (8) domains. Each item scored 1 to 7 (higher = better quality of life). MCID = 0.5 points.
4. **Nursing care adherence:** Purpose-designed questionnaire (0–100 points): <60 = non-adherence, 61–85 = adherence, 86–100 = full adherence.
5. **Caregiver psychological status:** Depression-anxiety-stress scale-21 (DASS-21) with depression,^[16]^ anxiety, and stress subscales (7 items each, scored 0–3; higher = more severe symptoms).
6. **Family functioning:** Family assessment device (FAD) measuring emotional communication, problem-solving, and family rules through 30 items (scored 1–4, total 30–120; higher = healthier functioning).^[17]^
7. **Clinical outcomes:** Number of asthma attacks (defined as acute worsening requiring systemic corticosteroids), emergency room visits, hospitalizations, and relapse rates (defined as return of symptoms requiring treatment escalation within 30 days of previous episode).

### 2.6. Interventions

#### 2.6.1. Control group (CG)

Participants in the CG received conventional treatment and care according to standard clinical guidelines. This included appropriate pharmacotherapy (inhaled corticosteroids, bronchodilators, and other medications as clinically indicated), routine monitoring of symptoms and pulmonary function, and standard patient education. Medical staff provided explanations of conditions and treatment plans to children and parents and addressed questions during regular clinic visits.

#### 2.6.2. Research group (RG)

The RG received identical conventional treatment as the CG, supplemented by a comprehensive management strategy combining gratitude-based positive psychology interventions and peer interaction education:

#### 2.6.3. Gratitude-based positive psychology components

1. *Personnel preparation:* A dedicated team comprising one pediatric nurse manager and 5 pediatric charge nurses underwent intensive training in gratitude-based intervention techniques. The training encompassed theoretical foundations and practical applications, with all team members completing the program and passing competency assessments.
2. *Hospital-based interventions:* Inspirational symposia conducted on admission day or the following day; Daily bedside sessions (10 minutes each) including positive memory exercises; Gratitude contemplation and expression sessions; Life essays activities guiding documentation of positive experiences; Hobby support providing resources for preferred activities.
3. *Home-based interventions:* Daily morning social media group announcements encouraging gratitude-based activities; Evening gratitude diary entries; Hobby cultivation with physical activity guidance specific to asthma; Twice-weekly educational content on asthma management; Monthly individualized follow-up communications.

#### 2.6.4. Peer interaction education components

Peer interaction sessions were facilitated by trained pediatric nurses (1:8 adult-to-child ratio), conducted in age-banded groups of 6 to 8 children (6–10 years and 11–14 years separately), meeting daily during hospitalization (30-minute sessions) and monthly thereafter (60-minute sessions). Attendance was tracked, with mean attendance of 82%. Sessions included: Collaborative knowledge exchange through heuristic questioning; Shared experiences of rhinitis and asthma symptoms; Reciprocal instruction in inhalation devices and peak flow meter usage; Mutual symptom monitoring and assistance; Collaborative symptom recognition and management.

The intervention period lasted 12 months for both groups, with discharged children reconvening monthly for continuation of the program.

### 2.7. Statistical analysis

Data were analyzed using IBM SPSS Statistics 26.0 and GraphPad Prism 8. Normality was tested for all measurement data. Baseline comparability was assessed using standardized mean differences (SMD) with target <0.1.

For Phase 1, univariate ANOVA identified variables potentially related to asthma control. Variables significant in univariate analysis (*P* < .05) were included in multivariate logistic regression to identify independent risk factors.

For Phase 2, linear mixed-effects models were used for repeated continuous outcomes (ACT, PAQLQ, FAD, DASS-21) with time, group, and group × time interaction as fixed effects and subject as random effect. Negative binomial regression was used for count outcomes (attacks, ER visits, hospitalizations) with results presented as incidence rate ratios (IRRs) with 95% confidence intervals (CIs). To address multiplicity, we used the Benjamini–Hochberg procedure to control false discovery rate at 0.05 across secondary endpoints. Propensity score adjustment was conducted for key baseline variables (age, gender, disease duration, presence of allergic rhinitis, and baseline psychological measures). Intent-to-treat analysis was employed using the last observation carried forward method for missing data, though no dropouts occurred. A *P* value <.05 was considered statistically significant for all analyses.

## 3. Results

### 3.1. Risk factors for poor asthma control

Phase 1 analysis of 152 children with bronchial asthma (102 fully controlled, 50 poorly controlled) revealed significant differences between CGs across multiple domains. As shown in Table 1, univariate analysis demonstrated that children with fully controlled asthma were significantly older (12.34 ± 3.17 vs 11.09 ± 4.28 years, *P* = .044, SMD = 0.33) and exhibited higher rates of medication adherence (67.65% vs 42.00%, *P* = .003, SMD = 0.53) compared to the poorly controlled group. The fully controlled group also showed significantly higher participation in health education (78.43% vs 56.00%, *P* = .004, SMD = 0.48), physical activity (71.57% vs 52.00%, *P* = .002, SMD = 0.40), and regular follow-up attendance (88.24% vs 68.00%, *P* = .003, SMD = 0.50).

**Table 1:** Factors associated with asthma control among children treated from June 2022 to June 2023.

Parameter	Fully controlled group (n = 102)	Poorly controlled group (n = 50)	SMD	χ^2^(*t*)	*P*
Age (yr)	12.34 ± 3.17	11.09 ± 4.28	0.33	2.028	.044*
Place of residence, n (%)					
Town	66 (64.71)	27 (54.00)	0.22	1.619	.203
Village	36 (35.29)	23 (46.00)			
Gender (female), n (%)	58 (56.86)	30 (60.00)	0.06	0.136	.713
Obesity, n (%)	18 (17.65)	6 (12.00)	0.16	0.805	.370
Disease duration (yr)	3.87 ± 4.69	4.15 ± 4.22	0.06	0.357	.722
Allergic rhinitis, n (%)	41 (40.20)	31 (62.00)	0.44	6.398	.011*
Primary caregivers (parents), n (%)	53 (51.96)	23 (46.00)	0.12	0.477	.490
Guardian smoking, n (%)	18 (17.65)	20 (40.00)	0.51	8.941	.003*
Medication adherence, n (%)	69 (67.65)	21 (42.00)	0.53	9.138	.003*
Family history of asthma, n (%)	8 (7.84)	9 (20.00)	0.35	4.65	.030*
DASS-21 score (points)					
Depression	17.05 ± 2.87	18.3 ± 3.1	0.42	3.086	.002*
Anxiety	17.92 ± 2.94	19.34 ± 2.81	0.49	2.457	.020*
Stress	16.53 ± 2.62	17.96 ± 3.28	0.48	2.904	.004*
FAD score (points)†	89.26 ± 11.64	80.68 ± 10.66	0.77	4.387	<.001*
Health education, n (%)	80 (78.43)	28 (56.00)	0.48	8.208	.004*
Physical activity, n (%)	73 (71.57)	26 (52.00)	0.40	9.300	.002*
Regular follow-up, n (%)	90 (88.24)	34 (68.00)	0.50	9.142	.003*

DASS-21 = depression-anxiety-stress scale-21, FAD = family assessment device, SMD = standardized mean difference.

† Higher FAD scores indicate better family functioning. SMD = standardized mean difference.

* *P* < .05 indicates statistical significance.

Conversely, the poorly controlled group demonstrated higher prevalence of comorbid allergic rhinitis (62.00% vs 40.20%, *P* = .011, SMD = 0.44), guardian smoking (40.00% vs 17.65%, *P* = .003, SMD = 0.51), and family history of asthma (20.00% vs 7.84%, *P* = .030, SMD = 0.35). Caregivers of poorly controlled children exhibited significantly higher psychological distress across all DASS-21 dimensions (depression: 18.3 ± 3.1 vs 17.05 ± 2.87, *P* = .002, SMD = 0.42; anxiety: 19.34 ± 2.81 vs 17.92 ± 2.94, *P* = .020, SMD = 0.49; stress: 17.96 ± 3.28 vs 16.53 ± 2.62, *P* = .004, SMD = 0.48). Additionally, family functioning scores were significantly lower in the poorly controlled group (80.68 ± 10.66 vs 89.26 ± 11.64, *P* < .001, SMD = 0.77).

Multivariate logistic regression analysis (Table 2) identified ten independent risk factors for poor asthma control: comorbid allergic rhinitis (odds ratio (OR) = 1.23, 95% CI: 0.98–1.67, *P* = .037), guardian smoking (OR = 1.36, 95% CI: 1.02–2.17, *P* = .03), medication non-adherence (OR = 1.44, 95% CI: 1.12–2.37, *P* = .022), family history of asthma (OR = 1.04, 95% CI: 0.90–1.89, *P* = .041), anxiety (OR = 1.89, 95% CI: 1.37–2.79, *P* = .038), stress (OR = 3.66, 95% CI: 1.16–8.93, *P* = .04), low family functioning scores (OR = 4.59, 95% CI: 1.12–9.33, *P* = .009), absence of health education (OR = 5.13, 95% CI: 1.54–7.80, *P* = .006), physical inactivity (OR = 2.28, 95% CI: 1.22–5.38, *P* = .045), and irregular follow-up (OR = 2.96, 95% CI: 1.17–6.52, *P* = .032).

**Table 2:** Multivariable logistic regression analysis of factors associated with poorly controlled asthma.

Variable	β	Wald χ^2^	*P*	OR	95% CI
Young age	1.57	3.874	.053	1.81	0.99–3.31
Combined allergic rhinitis†	0.276	4.443	.037*	1.23	0.98–1.67
Guardian smoking†	1.04	4.287	.03*	1.36	1.02–2.17
Medication non-adherence‡	1.29	5.152	.022*	1.44	1.12–2.37
Family history of asthma	0.665	4.214	.041*	1.04	0.90–1.89
Depression	1.2	2.38	.121	1.23	0.89–1.84
Anxiety	1.429	4.884	.038*	1.89	1.37–2.79
Stress	1.298	4.23	.04*	3.66	1.16–8.93
Low FAD score§	1.582	6.87	.009*	4.59	1.12–9.33
No health education¶	1.74	7.52	.006*	5.13	1.54–7.80
No physical activity	1.326	4.08	.045*	2.28	1.22–5.38
Irregular follow-up	1.427	4.61	.032*	2.96	1.17–6.52

CI = confidence interval, FAD = family assessment device, OR = odds ratio.

† Combined allergic rhinitis: presence of both asthma and allergic rhinitis.

‡ Medication non-adherence: <60% adherence to prescribed medications.

§ Low FAD score: <75 points on family assessment device.

¶ No health education: absence of structured asthma education program participation.

* *P* < .05 indicates statistical significance.

### 3.2. Baseline characteristics of intervention groups

The 120 children enrolled in Phase 2 were allocated equally to the RG and CG. Despite the non-randomized allocation, baseline demographic and clinical characteristics were comparable between groups with all SMDs < 0.1, as detailed in Table 3. There were no statistically significant differences in age (RG: 11.94 ± 2.81 vs CG: 11.37 ± 2.52 years, *P* = .396, SMD = 0.08), gender distribution (female: RG 58.33% vs CG 55.00%, *P* = .713, SMD = 0.07), disease duration (RG: 3.79 ± 4.15 vs CG: 3.52 ± 4.06 years, *P* = .719, SMD = 0.07), or prevalence of allergic rhinitis (RG: 51.67% vs CG: 45.00%, *P* = .465, SMD = 0.09). Similarly, psychological parameters (DASS-21 scores) and family functioning (FAD scores) showed no significant between-group differences at baseline, supporting the validity of group comparisons despite the non-randomized design. All 120 participants completed the 12-month study period with no dropouts reported.

**Table 3:** Clinical characteristics of patients in control and research groups.

Parameter	Control group (n = 60)	Research group (n = 60)	SMD	χ^2^(*t*)	*P*
Age (yr)	11.37 ± 2.52	11.94 ± 2.81	0.08	0.851	.396
Place of residence, n (%)					
Town	36 (60.00)	32 (53.33)	0.09	0.543	.461
Village	24 (40.00)	28 (46.67)			
Gender (female), n (%)	33 (55.00)	35 (58.33)	0.07	0.136	.713
Obesity, n (%)	8 (13.33)	11 (18.33)	0.09	0.563	.453
Disease duration (yr)	3.52 ± 4.06	3.79 ± 4.15	0.07	0.36	.719
Allergic rhinitis, n (%)	27 (45.00)	31 (51.67)	0.09	0.534	.465
Primary caregivers (parents), n (%)	26 (43.44)	30 (50.00)	0.08	0.536	.464
Guardian smoking, n (%)	20 (33.33)	16 (26.67)	0.07	0.635	.426
Family history of asthma, n (%)	5 (8.33)	7 (11.67)	0.06	0.37	.543
DASS-21 score (points)					
Depression	17.93 ± 3.34	18.12 ± 3.28	0.06	0.314	.754
Anxiety	18.27 ± 3.68	18.12 ± 3.11	0.04	0.241	.810
Stress	17.18 ± 3.41	16.97 ± 3.76	0.06	0.321	.749
FAD score (points)	80.11 ± 11.65	80.37 ± 10.87	0.02	0.126	.900

DASS-21 = depression-anxiety-stress scale-21, FAD = family assessment device, SMD = standardized mean difference.

All SMDs < 0.1 indicating good balance between groups.

### 3.3. Intervention effects on primary outcome: self-management abilities

**Figure 1.**
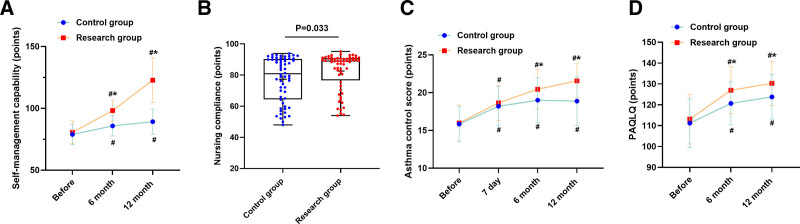
Comparison of treatment adherence and clinical outcomes between CG and RG after 12 mo. (A) Self-management ability scores showing significant improvement in the RG at both 6 and 12 mo. (B) Treatment adherence demonstrating higher overall adherence in the RG (90% vs 76.7%, *P* = .033), with more children achieving full adherence (n = 37 vs n = 26) and fewer cases of non-adherence (n = 6 vs n = 14). (C) ACT scores indicating better control in the RG at 6 mo (horizontal line indicates ACT = 20 threshold for controlled asthma). (D) PAQLQ scores showing enhanced quality of life in the RG (horizontal dashed line indicates MCID of 0.5). *P* < .05 compared with CG; #*P* < .05 compared with baseline assessment. Error bars represent 95% confidence intervals. ACT = asthma control test, CG = control group, MCID = minimum clinically important difference, PAQLQ = pediatric asthma quality of life questionnaire, RG = research group.

Self-management abilities, the primary outcome, showed significant improvement in both groups over time, with superior outcomes in the RG (Fig. 1A). The adjusted mean difference between groups was 8.4 points (95% CI: 4.2–12.6) at 6 months and 11.2 points (95% CI: 6.8–15.6) at 12 months (both *P* < .001), indicating progressive enhancement of self-management capabilities with sustained intervention.

### 3.4. Treatment adherence and asthma control

Analysis of nursing care adherence following the 12-month intervention revealed significant between-group differences (*P* = .033, Fig. 1B). The RG demonstrated substantially higher overall adherence rates compared to the CG (90.00% vs 76.67%). Full adherence was achieved by 61.67% in RG versus 43.33% in CG, with lower rates of non-adherence (RG: 10.00% vs CG: 23.33%).

Asthma control improved significantly in both groups, with the RG achieving higher ACT scores at 6 months (adjusted mean difference: 2.3 points, 95% CI: 1.1–3.5, *P* = .049; Fig. 1C). At 12 months, 75% of RG achieved controlled asthma (ACT ≥ 20) versus 58% of CG (*P* = .046).

### 3.5. Quality of life and family functioning

**Figure 2.**
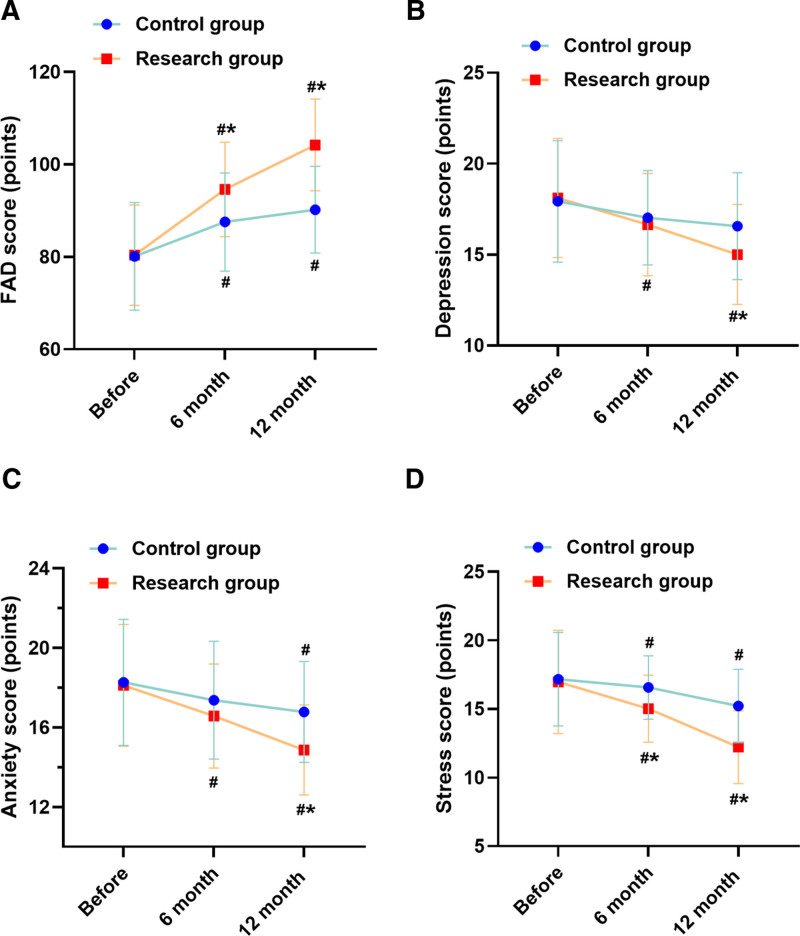
Psychosocial outcomes in families of children receiving CG versus RG interventions. (A) FAD scores showing improved family functioning in the RG. (B) Caregiver depression scores from DASS-21 assessment. (C) Caregiver anxiety scores demonstrating significant reduction in the RG at 12 mo. (D) Caregiver stress scores showing earlier improvement in the RG at 6 mo. *P < .05 compared with CG; #*P* < .05 compared with baseline assessment. Higher FAD scores indicate better family functioning; lower DASS-21 scores indicate improved psychological status. Error bars represent 95% confidence intervals. CG = control group, DASS-21 = depression-anxiety-stress scale-21, FAD = family assessment device, RG = research group.

Quality of life assessments showed the RG achieving significantly higher PAQLQ scores at 6 months (adjusted mean difference: 0.6 points, 95% CI: 0.3–0.9, *P* = .018; Fig. 1D), exceeding the MCID of 0.5 points. Family functioning improved substantially in the RG with significantly greater enhancement in FAD scores at 6 months compared to control (adjusted mean difference: 7.8 points, 95% CI: 3.2–12.4, *P* = .004; Fig. 2A).

### 3.6. Caregiver psychological status

Caregiver psychological parameters showed differential improvement patterns. Depression scores decreased significantly more in the RG at twelve months (adjusted mean difference: -1.8 points, 95% CI: −-3.2 to −0.4, *P* = .048; Fig. 2B). Anxiety reduction was greater in RG at twelve months (adjusted mean difference: −2.1 points, 95% CI: −3.6 to −0.6, *P* = .003; Fig. 2C). Stress levels showed earlier response with significant between-group differences at 6 months (adjusted mean difference: −1.6 points, 95% CI: −2.9 to −0.3, *P* = .045; Fig. 2D).

### 3.7. Clinical outcomes and healthcare utilization

Analysis using negative binomial regression revealed consistently superior outcomes in the RG (Table 4). The RG experienced significantly lower rates of asthma attacks (IRR = 0.85, 95% CI: 0.74–0.98, *P* < .001), emergency room visits (IRR = 0.74, 95% CI: 0.62–0.89, *P* < .001), and hospitalizations (IRR = 0.77, 95% CI: 0.65–0.91, *P* < .001). The overall relapse rate showed substantial reduction (IRR = 0.33, 95% CI: 0.12–0.91, *P* = .032).

**Table 4:** Clinical outcomes in children receiving control versus research group interventions over 12 mo.

Outcome	Control group (n = 60)	Research group (n = 60)	IRR (95% CI)	*P*
Asthma attacks (rate per person-year)	3.48	2.97	0.85 (0.74–0.98)	<.001
Emergency visits (rate per person-year)	2.65	1.97	0.74 (0.62–0.89)	<.001
Hospitalizations (rate per person-year)	3.01	2.31	0.77 (0.65–0.91)	<.001
Relapse rate, n (%)	12 (20.00)	4 (6.67)	0.33 (0.12–0.91)	.032

CI = confidence interval, IRR = incidence rate ratio.

CI relapse defined as return of symptoms requiring treatment escalation within 30 days of previous episode.

## 4. Discussion

This integrated approach yielded clinically meaningful improvements, with ACT changes approaching the MCID of 3 points and PAQLQ exceeding 0.5, suggesting practical benefits in control and quality of life. Our findings suggest that this integrated approach was associated with improved children’s self-management abilities, which in turn enhanced care adherence, relieved caregivers’ negative emotions, and improved family functioning, ultimately leading to better asthma control.

In recent years, some studies in China have explored the application of gratitude-based positive psychology and peer interaction education in asthma management, revealing that both strategies can improve outcomes for children with asthma. However, there are relatively few studies examining these approaches, and to our knowledge, no previous reports have evaluated these strategies used in conjunction. Similarly, there are relatively few international studies on the application of gratitude-based positive psychology in disease management, though beneficial effects on quality of life have been reported in patients with conditions such as bladder cancer.^[13]^ Overall, the integration of gratitude-based positive psychology and peer interaction education represents a gap in both domestic and international research that warrants further exploration.

As lifestyles change, the factors influencing asthma control also evolve. The first phase of our study identified several key risk factors for poor asthma control, including comorbid allergic rhinitis (consistent with established links), guardian smoking, medication non-adherence, family history of asthma, anxiety, stress, low family functioning scores, absence of health education, physical inactivity, and irregular follow-up.

Previous research has emphasized that self-management education for children and guardians is essential.^[18]^ Health education, healthy lifestyle activities, and effective family communication are all integral components of self-management education. In our retrospective analysis, health education (*P* = .006) and family functional health (*P* = .009) showed the strongest statistical associations with asthma control. Health education can help children and parents understand appropriate physical exercise methods and reasonable dietary structures, improve treatment adherence, and reduce acute asthma exacerbations.

Environmental factors also play important roles in asthma management. For asthmatics, certain substances in tobacco can act as direct allergens, triggering asthma attacks, and in severe cases, contribute to chronic bronchitis. Thus, guardian smoking is detrimental to asthma control in children.^[19,20]^ Caregiver anxiety and stress were also identified as risk factors exacerbating asthma flare-ups in children, consistent with findings from Margolis et al.^[21]^ Our integrated care approach in the prospective phase was designed to address these risk factors by implementing a comprehensive management strategy for school-aged children with asthma.

Our prospective intervention study found significantly higher care adherence in the RG compared to the CG. Since asthma is a chronic disease requiring long-term continuous treatment and management, adherence to medical advice is crucial. Non-adherence can lead to exacerbation of asthma symptoms with adverse effects. The integrated management approach in our study-which included inspirational lectures, regular group interactions, and peer support – appeared to enhance the motivation of children with asthma, improve knowledge, and increase care adherence. Jia et al reported that certain parenting styles can improve medication adherence and asthma control in children,^[22]^ which aligns with our findings that improved care adherence has beneficial effects on asthma outcomes.

Children with asthma must endure physical discomfort caused by the disease for extended periods, and long-term medication may increase their psychological burden, affecting physical and mental health and reducing quality of life. Our prospective evaluation found significant improvements in PAQLQ scores among children receiving the enhanced management program. This finding is consistent with a pilot study from Thailand, which reported that an educational intervention for caregivers improved children’s PAQLQ scores and enhanced asthma management.^[23]^ There is also evidence of a significant correlation between PAQLQ and ACT scores in children with asthma undergoing treatment, with parallel increases in both measures.^[24]^ The ACT scores in our study showed a pattern similar to the PAQLQ findings, with significantly higher scores in the RG compared to the CG at 6 months of intervention (*P* = .049). These results suggest that the integrated approach combining gratitude-based positive psychology with peer interaction education can improve children’s self-management skills, leading to better asthma control and quality of life.

Caregivers play a crucial role in the self-management of children with asthma, and the family environment significantly impacts the health of children with asthma over extended periods. Changes in family functioning may affect disease management, medication adherence, and psychological well-being of children with asthma. A meta-analysis showed that the well-being of children with chronic diseases is closely related to family functioning, and improving family functioning is key to influencing disease outcomes.^[25]^ In our study, the integrated management program was associated with improved family functioning, including aspects such as communication effectiveness and problem-solving skills.

The gratitude-based positive psychology emphasizes the positive impact of gratitude and positive cognition on individuals and families. Family-centered care may lead to the restructuring and reshaping of family functioning through promoting understanding, support, and emotional connections among family members. Strengthened family functioning can better address the challenges posed by pediatric asthma and provide more comprehensive medical and emotional support to children and caregivers. This approach may help families view asthma-related challenges from a perspective of gratitude and improve perceptions and attitudes toward asthma treatment through cognitive restructuring.

Furthermore, our integrated management approach was associated with reduced negative emotions such as anxiety and stress in primary caregivers (*P* < .05), thus improving overall family functioning. We observed significant decreases in anxiety, depression, and stress among caregivers after the intervention, which may be attributed to the gratitude-based positive psychology component guiding asthmatic children and their caregivers to view life and disease treatment through an attitude of gratitude and positive cognitive approaches. This perspective may help caregivers experience gratitude and positive emotions, improving their overall emotional state. Moreover, children’s improved self-management skills and better asthma control likely contributed to a favorable cycle of positive emotions. In summary, our findings suggest that the integrated approach was associated with improved caregivers’ negative emotions, increased family functional health, and had beneficial effects on children’s disease control. The frequency of relapses and relapse rates within 12 months were also significantly reduced in the RG, further supporting the effectiveness of this approach.

Several limitations should be considered when interpreting our findings. First, as a non-randomized prospective study, the time-based allocation process used in our intervention phase may have introduced selection bias despite the comparable baseline characteristics between groups. Seasonality risk from time-based allocation, potential contamination, and omission of clinical predictors in Phase 1 are additional limitations. Second, while we used propensity score adjustment to account for potential confounding factors, unmeasured confounders could still have influenced our results. Third, the single-center design limits the generalizability of our findings to other settings or populations. Fourth, although we documented care program components, implementation fidelity could not be fully standardized across all participants. Fifth, while assessors were blinded to group allocation, the nature of the intervention made it impossible to blind participants and care providers, potentially introducing performance bias. Finally, while our follow-up period was 12 months, longer-term outcomes remain unknown. Future studies employing full randomization with cluster design would help confirm our findings and minimize contamination between intervention groups.

## 5. CONCLUSION

This study provides evidence for the effectiveness of an integrated clinical approach combining gratitude-based positive psychology with peer interaction education in managing school-aged children with asthma. Through our two-phase design-first identifying risk factors and then prospectively evaluating a targeted intervention-we developed an evidence-based management strategy that significantly improved children’s self-management abilities, treatment adherence, and family functional health, which in turn reduced asthma relapses and improved children’s quality of life. Our findings enrich the understanding of nursing interventions for children with asthma and suggest a potentially valuable clinical approach for improving the quality of life of children with asthma. Future prospective randomized studies are warranted to further validate these findings and explore the long-term sustainability of these benefits.

## Acknowledgments

The authors would like to thank the nursing staff of the Pediatric Department at No. 980 Hospital of the Joint Logistics Support Force for their dedication to implementing the enhanced care program. We also extend our gratitude to all the children and families who participated in this study. Special thanks to the hospital administration for supporting innovative approaches to pediatric asthma management. All individuals named in this Acknowledgments have provided permission to be acknowledged.

## Author contributions

**Conceptualization:** Haixin Chen, Lijin Gao, Lijia Liu, Zhou Junli, Wen Zhang.

**Data curation:** Haixin Chen, Lijin Gao, Lijia Liu, Zhou Junli, Wen Zhang.

**Formal analysis:** Haixin Chen, Junping Du, Wen Zhang.

**Funding acquisition:** Xiuming Wei, Wen Zhang.

**Investigation:** Xiuming Wei, Miaomiao Chen, Zhou Junli, Wen Zhang.

**Methodology:** Xiuming Wei, Lijia Liu, Miaomiao Chen, Junping Du, Wen Zhang.

**Project administration:** Wen Zhang.

**Resources:** Junping Du, Wen Zhang.

**Software:** Lijin Gao, Lijia Liu, Miaomiao Chen, Zhou Junli, Junping Du, Wen Zhang.

**Supervision:** Wen Zhang.

**Validation:** Xiuming Wei, Lijia Liu, Miaomiao Chen, Junping Du, Wen Zhang.

**Visualization:** Lijin Gao, Miaomiao Chen, Junping Du, Wen Zhang.

**Writing – original draft:** Haixin Chen, Wen Zhang.

**Writing – review & editing:** Haixin Chen, Xiuming Wei, Lijia Liu, Zhou Junli, Junping Du, Wen Zhang.
